# Moving average procedures as an additional tool for real-time analytical quality control: challenges and opportunities of implementation in small-volume medical laboratories

**DOI:** 10.11613/BM.2022.010705

**Published:** 2021-12-15

**Authors:** Vera Lukić, Svetlana Ignjatović

**Affiliations:** 1 Department of Laboratory Diagnostics, Railway Healthcare Institute, Belgrade, Serbia; 2 Department of Medical Biochemistry, University of Belgrade, Faculty of Pharmacy, Belgrade, Serbia; 3 Center for Medical Biochemistry, Clinical Center of Serbia, Belgrade, Serbia

**Keywords:** quality control, moving average, patient-based real-time quality control, laboratory information system

## Abstract

**Introduction:**

Moving average (MA) is one possible way to use patient results for analytical quality control in medical laboratories. The aims of this study were to: (1) implement previously optimized MA procedures for 10 clinical chemistry analytes into the laboratory information system (LIS); (2) monitor their performance as a real-time quality control tool, and (3) define an algorithm for MA alarm management in a small-volume laboratory to suit the specific laboratory.

**Materials and methods:**

Moving average alarms were monitored and analysed over a period of 6 months on all patient results (total of 73,059) obtained for 10 clinical chemistry parameters. The optimal MA procedures were selected previously using an already described technique called the bias detection simulation method, considering the ability of bias detection the size of total allowable error as the key parameter for optimization.

**Results:**

During 6 months, 17 MA alarms were registered, which is 0.023% of the total number of generated MA values. In 65% of cases, their cause was of pre-analytical origin, in 12% of analytical origin, and in 23% the cause was not found. The highest alarm rate was determined on sodium (0.10%), and the lowest on calcium and chloride.

**Conclusions:**

This paper showed that even in a small-volume laboratory, previously optimized MA procedures could be successfully implemented in the LIS and used for continuous quality control. Review of patient results, re-analysis of samples from the stable period, analysis of internal quality control samples and assessment of the analyser malfunctions and maintenance log have been proposed for the algorithm for managing MA alarms.

## Introduction

Internal quality control is a critical segment of medical laboratory practice. It is based on analysing commercially available control materials at specific time intervals (*1*). The results of these measurements are compared with known target values and standard deviations, following well-established guidelines and recommendations (*2*). This type of control is adequately supported by modern automated analysers and information systems. However, the scientific community is aware that this traditional type of quality control has its weaknesses (*3*). These are, first of all, its intermittency, and the problem of commutability (*4*). Due to the intermittency of performing traditional quality control, there is a risk that the analytical bias that occurs between two control measurements will remain undetected leading to the release of erroneous results in this period (*2*, *5*). In addition, the potential non-commutability between control materials and real patient samples compromises the sensitivity and specificity of this type of control (*5*). Therefore, there is a need to introduce additional control mechanisms that could overcome these flaws and provide continuous monitoring of the analytical process.

Patient-based real-time quality control (PBRTQC) can be used for this purpose. One of the possible ways of using patient results for analytical quality control is the moving average (MA) (*2*). Moving average is the calculated average value of a parameter based on a series of patient results. This average value is continuously recalculated every time a new patient result is received from the analyser. To establish a MA control procedure, it is necessary to define a number of parameters for each analyte in every individual laboratory: inclusion criteria, calculation formula, block size or weighting factor (depending on the formula) and control limits (*6*). The complexity of locally defining optimal MA procedures has been a major obstacle to the wider use of MA in medical laboratories in previous decades (*7*). In recent years, thanks to the ability of some types of software to provide support in the calculations, the idea of the MA method has regained relevance (*8*).

The concept of MA quality control is attractive because it enables continuous quality control of laboratory work that takes place simultaneously with the analysis of patient samples (*9*, *10*). However, there are many unresolved issues regarding this control concept. The most significant problems concern the local determination of optimal MA procedures for each analyte, the potential of the existing laboratory information system (LIS) for MA implementation, lack of guidelines for dealing with situations when MA control is out of acceptable limits, and the impact of laboratory testing volume (daily number of samples and required tests) on the effectiveness of this form of control (*2*). The formation of the Working group on patient-based real-time quality control by the International Federation of Clinical Chemistry and Laboratory Medicine (IFCC) has further emphasized the importance of this topic at the global level (*11*).

The aims of this study were to: (*1*) implement previously optimized MA procedures for 10 clinical chemistry analytes into the laboratory information system (LIS); (*2*) monitor their performance as a real-time quality control tool, and (*3*) define an algorithm for MA alarm management in a small-volume laboratory to suit the specific laboratory. According to currently available literature data, this is the first time that PBRTQC has been implemented as additional quality control for clinical chemistry tests in a medical laboratory in Serbia.

## Materials and methods

### Materials

The study was designed as retrospective data collection from the LIS (Next lab, BitImpex, Belgrade, Serbia). It was conducted from July to December 2019 at the Department of laboratory diagnostics, Railway Healthcare Institute, Belgrade, Serbia. The laboratory operates at the primary level of healthcare, serving only outpatients from the general adult population and performing about 400,000 tests annually. Monitoring of the occurrence of MA alarms and their analysis were done on all patient results (a total of 73,059) obtained during the mentioned period of 6 months for the following 10 clinical chemistry analytes: albumin, aspartate aminotransferase (AST), creatinine, calcium, chloride, HDL (high-density lipoprotein) cholesterol, potassium, sodium, and total protein. These 10 analytes were selected based on two criteria. The first criterion was the daily number of performed tests. AST, creatinine, cholesterol, and HDL-cholesterol were selected as representatives of high-frequency tests, sodium and potassium as moderately frequent, and chloride, calcium, total protein, and albumin as low-frequency tests in our laboratory. The other reason why we chose these 10 analytes is that MA procedures have been optimized for them that can detect clinically significant bias within the daily number of tests. Previously, the optimal MA procedures were selected from the total number of 87,092 patient results of the 10 listed clinical chemistry analytes extracted from the LIS for the period January to June 2018. Only results without interference from haemolysis, icterus and lipemia were included. During this period, both internal and external quality control were within acceptable limits for all ten analytes. Calibrations and quality control procedures were regularly performed according to an internal laboratory protocol (which is in line with test manufacturer recommendations).

Considering the time window between the optimization of MA procedures and the start of this study, and the rationalization made in the meantime that further reduced the daily volume of testing, we checked the established control limits on a total number of 37,008 results of the 10 studied analytes for a period of 3 months (March to May 2019) and found that no corrections were required.

All tests were performed on the Architect c16000 (Abbott, Abbott Park, USA) with the original reagents. The use of data from the LIS for this study was approved by the Ethical Committee of the Railway Healthcare Institute, Belgrade, Serbia.

### Methods

Selection of the optimal MA procedure for each of the 10 examined clinical chemistry analytes was previously done using the bias detection simulation method described by van Rossum (*7*, *12*, *13*). The bias detection simulation method comprises the examination of different combinations of MA procedure parameters for each analyte and the ability of each examined MA procedure to detect biases of different sizes through dedicated software. We have previously described in detail the process of selecting, optimizing, and validating MA procedures on the example of creatinine, potassium, sodium, and albumin (*12*). Respectively, the same was done for the other six analytes. In this study, we used these previously obtained data for implementing MA procedures into the LIS.

In brief, optimization and validation of MA procedures were done as follows: for each MA procedure, a choice was made of the inclusion criteria (truncation limit), calculation formula (simple MA or exponentially weighted MA – EWMA), block size or weighting factor (depending on the formula), and control limits. Except Bull’s algorithm, mainly used on haematological analysers, the most commonly used algorithms for calculating MA values are simple MA and EWMA. In the process of choosing optimal MA procedures, we examined both simple MA and EWMA formulas for each analyte. Simple MA is calculated using the formula: z _(t)_ = x _(t)_ / n + x _(t - 1)_ / n + x _(t - 2)_ / n + ... + x _(t - n + 1)_ / n, where z _(t)_ is the calculated mean value on the result number t, x is the result, and n is the block size. The size of the block is the number of consecutive test results that are used for calculating an MA value in a simple MA algorithm. Exponentially weighted MA is calculated using the formula: z _(t)_ = λ x _(t)_ + (1 - λ) z _(t - 1)_, where z _(t)_ is the calculated mean value on the result number t, x is the result, and λ is the weighting factor. As the starting point for z_(t-1)_, the mean of the overall population was used. A weighting factor is a coefficient that determines how much current and previous test results affect calculation in the EWMA algorithm. It can take values between 0 and 1. The upper and lower control limits of each MA procedure were established as the maximum and the minimum value of the MA. In this way, false alarms should be almost completely avoided in routine practice. These minimum and maximum values were obtained for each combination of the calculation formula and truncation limits. In addition, they were calculated without truncation limits. Truncation limits are the endpoints of concentration ranges of examined analytes that are included in MA calculation. The choice of truncation limits depends on the patient population with which the laboratory is working. Based on the spread in the concentration values of examined analytes, different truncation limits were tested. For each of the 10 analytes, calculations were carried out first without the truncation limits, and then with them.

Then, a simulation was performed with the introduction of biases varying from - 50% to 50% into the results of all 10 analytes, including the bias equal to the total allowable error (TEa) for each analyte. Clinical Laboratory Improvement Amendments (CLIA) data for TEa were used (*14*). For tests for which CLIA does not give a percentage but an absolute value of TEa (calcium, potassium, and sodium), we used data from the work of Westgard *et al.* (*15*). The authors of this paper defined the percentage TEa for each of these tests at analyte concentrations most critical to medical decision making.

The obtained results of simulation were presented and analysed using the MA bias detection curves and MA validation charts previously described by van Rossum (*13*). An optimal combination of the formula, truncation limits, and control limits was chosen by the selection of optimal bias detection performance. The ability of an MA procedure to detect bias the size of the TEa within the daily number of tests performed in the laboratory was considered a key parameter for optimization, based on literature data (*2*, *8*, *9*, *16*). Moving average validation charts provide data about the number of results needed to detect a bias of a specific size. The median number of results required to detect a particular bias means that in 50% of cases, the bias will be detected in less than that number of results, and in 50% of cases, the detection will require more results. Additionally, the ability of an optimized MA procedure to detect bias the size of minimum TEa based on biological variation was read from MA validation charts. Minimum TEa was calculated using the formula: TE_a_ = 1.65 (0.75 x CV_i_) + 0.375 (CV_i_^2^ + CV_g_^2^)^1/2^, where CV_i_ is intraindividual variation and CV_g_ interindividual variation, for which data were taken from the EuBIVAS summary report (*17*). Due to the complexity of calculations that are necessary if a laboratory decides to use this type of quality control, a software is needed which performs all the calculations for each MA procedure, then allows their comparison to select the optimal one, and also provides the possibility of MA procedure validation (*4*, *18*). In our study, all MA calculations and simulations, as well as optimization and validation of MA procedures, were performed with the MA Generator software (Huvaros B.V., Bloemendaal, The Netherlands) (*19*).

Next, the optimized MA procedures were implemented into the LIS. First, the program was set to define which form of sample ID (SID) would participate in the MA calculation, thus excluding all control sample measurements (both internal and external quality control). Secondly, the user was allowed to enter in the LIS the following parameters for each laboratory test: formula for MA calculation (simple MA or EWMA), lower and upper truncation limits, block size or weighting factor (depending on the formula), and lower and upper control limits. When receiving results from the analyser, the program first checks the form of the SID; if it is inappropriate, the result will not be included in the MA procedure. The value from the analyser is then compared with the given truncation limits; if it is outside the truncation limits, again, the result will not enter the MA calculation. If the result meets the criteria for inclusion in the MA procedure, the program calculates the MA value for the current result. For each new result received from the analyser, the MA value is recalculated. If the calculated MA value for an analyte is below the lower or above the upper control limit, the LIS generates an MA alarm. The MA alarm is a visual warning that the MA value calculated for an analyte is outside the specified control limits. The LIS also provides a table overview of MA values with the patient results that generated an alarm specifically marked, as well as a graph of all MA values for an individual analyte. The graph simultaneously shows patient results received from the analyser and the MA values calculated for each of them.

The functioning of the described software solution was first checked on a historical set of patient results from the previous 3 months, and no modifications were needed. After that, we started using it in real-time over a 6-month period. Regular laboratory work and the traditional quality control protocols were not disturbed because the entire MA concept in the LIS was visible to only one biochemist with the appropriate login data, while the other operators continued to follow the existing routine laboratory procedures. The performance of the 10 implemented MA procedures was assessed by monitoring the occurrence of MA alarms. The procedures used for MA alarm analysis included: a review of patient results, re-analysis of patient samples from a stable period, analysis of internal control samples, review of the analyser malfunctions and maintenance log. In the re-analysis of patient samples, a stable period was when there was no MA alarm for the analyte on the previous working day. As described by Liu *et al.*, we retested 3 patient samples from the stable period and determined there was an analytical shift if the difference between the original and the retested result was greater than 2 analytical standard deviations (obtained from quality control data from the analyser) in at least one of 3 retested samples (*20*). In serum samples stored under standard laboratory conditions (off clot or serum separator, at 2 to 8 °C), all the 10 studied analytes are stable for more than 72 hours, which is the maximum interval between two working days in our laboratory.

## Results

Table 1 shows the characteristics of selected MA procedures for 10 clinical chemistry analytes. The number of results needed to detect a bias equal to the TEa based on CLIA data (shown in Table 1 and Table 2) was obtained from MA validation charts during the validation of optimized MA procedures.

**Table 1:** Characteristics of optimized MA procedures for each of the 10 analytes and their capabilities for bias detection

**Analyte**	**Calculation algorithm**	**Truncation** **limit**		**Control limit**		**Average daily** **number of tests**	**TEa (%)**	**Number of results** **needed to detect** **a bias equal to the TEa**		
		**lower**	**upper**	**lower**	**upper**			**Minimum**	**Median**	**Maximum**
Albumin (g/L)	Mean Block size: 10	/	/	40	46	20	- 10	6	7	13
							+ 10	5	7	19
AST (U/L)	Mean Block size: 100	/	50	17	22	120	- 20	20	55	120
							+ 20	44	77	118
Calcium (mmol/L)	Mean Block size: 10	/	/	2.20	2.61	21	- 10	6	8	10
							+ 10	4	10	16
Chloride (mmol/L)	Mean Block size: 10	/	/	100	107	23	- 5	4	7	17
							+ 5	5	8	10
Cholesterol (mmol/L)	Mean Block size: 25	/	/	4.8	6.4	96	- 10	14	44	134
							+ 10	28	65	225
Creatinine (μmol/L)	EWMA Weighting factor: 0.1	/	150	62	90	103	- 15	6	20	44
							+ 15	27	86	360
HDL (mmol/L)	Mean Block size: 25	/	/	1.1	1.6	93	- 30	14	18	24
							+ 30	13	18	65
Potassium (mmol/L)	EWMA Weighting factor: 0.1	/	6.0	3.9	4.8	42	- 18	4	8	13
							+18	5	9	18
Sodium (mmol/L)	Mean Block size: 25	/	/	137	142	39	- 4	11	14	18
							+ 4	4	7	10
Total protein (g/L)	EWMA Weighting factor: 0.05	/	/	69	75	22	- 10	8	12	17
							+ 10	5	9	13
MA – moving average. EWMA – exponentially weighted moving average. TEa – total allowable error. CLIA data for TEa were used. AST - Aspartate aminotransferase. HDL - High-density lipoprotein cholesterol.										

**Table 2:** Ability of optimized MA procedures to detect minimum TEa based on biological variation within the daily number of tests

**Analyte**	**Average daily** **number of tests**	**MinimumTEa (%)** **based on biological** **variation**	**Number of results** **needed to detect** **a bias equal to the TEa**		
			**Minimum**	**Median**	**Maximum**
Albumin	20	- 5	8	18	69
		+ 5	7	16	61
AST	120	- 20	20	55	120
		+ 20	44	77	118
Cholesterol	96	- 10	14	44	134
		+ 10	28	65	225
Sodium	39	+ 1	14	25	180
Total protein	22	+ 5	7	21	68
MA – moving average. TEa – total allowable error. AST - Aspartate aminotransferase.					

**Figure 1:**
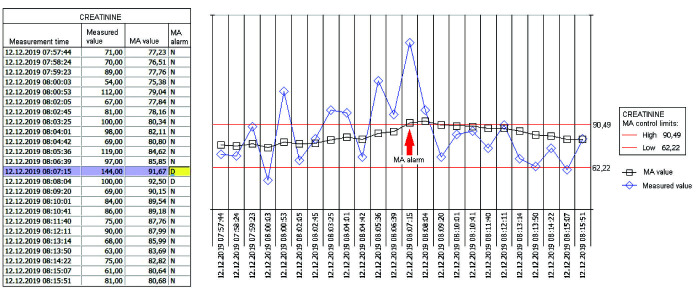
Table and graph presentation of an MA alarm for creatinine from the LIS. The values for control limits are expressed in μmol/L. In the table: the letter “D” means “Yes” and the letter “N” means “No”. MA – moving average.

**Figure 2:**
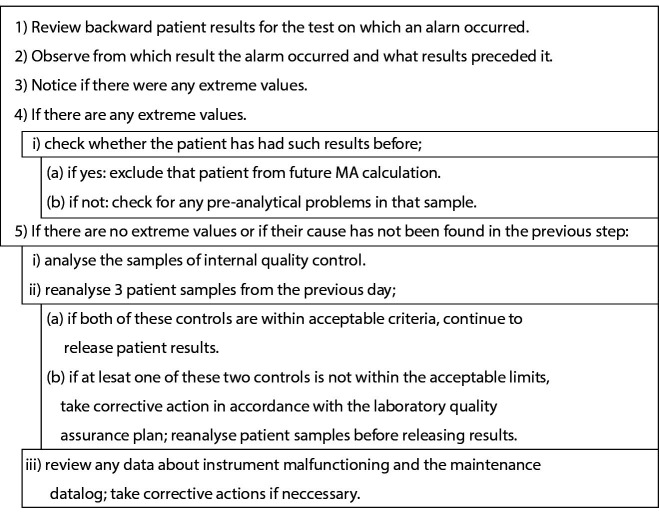
Algorithm for evaluation of moving average alarms.

Five out of ten optimized MA procedures were able to detect bias the size of TEa based on biological variation data, and they are shown in Table 2. For a period of 6 months, a total of 73,059 MA values were generated for the 10 biochemical analytes examined. We registered 17 MA alarms, which is 0.023% of the total number of MA values. The number of MA alarms *per* individual analyte is shown in Table 3. For every MA alarm, the LIS provided a table and graphical presentation that help to analyse the cause of the alarm, as shown in Figure 1 in the example of the MA alarm for creatinine. Every MA alarm was evaluated according to the algorithm shown in Figure 2.

**Table 3:** Moving average alarms *per* analyte

**Analyte**	**Number of generated MA values**	**Number of MA alarms**	**MA alarm rate (%)**
Albumin	2538	1	0.039
AST	15,095	2	0.013
Calcium	2667	0	0
Chloride	2894	0	0
Cholesterol	12,147	3	0.025
Creatinine	13,008	2	0.015
HDL	11,707	2	0.017
Potassium	5280	1	0.019
Sodium	4891	5	0.102
Total protein	2832	1	0.035
MA – moving average. AST - Aspartate aminotransferase. HDL - High-density lipoprotein cholesterol.			

Table 4 shows which algorithm steps were used to investigate each of the MA alarms and which alarm causes were detected. As part of the evaluation of alarms 3 and 5 for sodium, after recalibration, a re-analysis of all patient samples from that day was performed. The difference in sodium concentrations between the original and the retested results in any of the patients was not greater than 4 mmol/L, which is the CLIA defined value of TEa for this analyte.

**Table 4:** Work-up procedures and found causes of MA alarms

**Analyte**	**MA alarm**	**Work-up actions performed**					**Action performed regarding patient results**
		**Review of patient results**	**Observed preanalytical problem**	**Retesting of 3** **patient samples** **from the day before** **(1:2SD rule)**	**IQC** **(3 levels,** **1:2SD rule)**	**Maintenance** **and error log** **review**	
Albumin	1. Lower	Yes: 2 consecutive low results	Yes; Both patients are pregnant women	No	No	No	Results released
AST	1. Upper	Yes	No	Yes OK	Yes OK	Yes OK	Results released
	2. Upper	Yes: 3 consecutive results near the upper truncation limit	No	Yes OK	Yes OK	Yes OK	Results released
Cholesterol	1. Upper	Yes: 1 extremely high result	Yes: Patient has a history of extremely high cholesterol levels	No	No	No	Results released Patient excluded from future MA calculations
	2. Upper	Yes: 1 extremely high result	Yes: Patient has a history of extremely high cholesterol levels	No	No	No	Results released Patient excluded from future MA calculations
	3. Upper	Yes	No	Yes OK	Yes OK	Yes OK	Results released
Creatinine	1. Upper	Yes: 1 result near the upper truncation limit	Yes: Patient has a history of elevated creatinine levels	No	No	No	Results released Patient excluded from future MA calculations
	2. Upper	Yes: 3 results near the upper truncation limit	No	Yes OK	Yes OK	Yes OK	Results released
HDL	1. Lower	Yes: 1 low result	Yes: Patient has a history of extremely low HDL-cholesterol levels	No	No	No	Results released Patient excluded from future MA calculations
	2. Lower	Yes	No	Yes OK	Yes OK	Yes OK	Results released
Potassium	1. Lower	Yes: 1 low result repeated	No	No	No	No	Results released
Sodium	1. Upper	Yes: 1 high result	No	Yes OK	Yes OK	Yes OK	Results released
	2. Upper	Yes: 1 extremely high result	Yes: Sample diluted with saline	No	No	No	Test repeated on the original (undiluted) sample Results released
	3. Upper	Yes	No	Yes	Yes OK (but 0.5 SD upward shift)	Yes OK	Recalibration done Patient samples retested and new results issued
	4. Lower	Yes	No	Yes OK	Yes OK	Yes OK	Results released
	5. Lower	Yes	No	Yes	Yes OK (but 0.4 SD downward shift)	Yes OK	Recalibration done Patient samples retested and new results issued
Total protein	1. Upper	Yes: 1 extremely high result	Yes: Lipemic serum	No	No	No	New sample requested Results released
MA – moving average. IQC - internal quality control. SD – standard deviation. AST - Aspartate aminotransferase. HDL - High-density lipoprotein cholesterol.							

During the evaluation of an alarm for total protein, lipemia was found as the cause. Because our clinical chemistry analyser simultaneously transmits the results of ordered tests and HIL testing to the LIS, the occurrence of alarms caused by interferences could not be eliminated, but it can be easily revealed using the algorithm presented in Figure 2. Participation of individual groups of causes in the total number of MA alarms is illustrated in Table 5.

**Table 5:** Groups of detected causes of MA alarms

**Cause of MA alarm**	**Number of alarms**	**Percentage of the alarms total number (%)**
Abnormal patient result	9	53
Pre-analytical sample problem	2	12
Small analytical shift	2	12
No cause identified	4	23
MA – moving average.		

## Discussion

Our study highlighted the 3 key prerequisites for the routine use of the PBRTQC concept in medical laboratories: the availability of dedicated software for selecting optimal MA procedures, adequate implementation of MA in LIS, and the existence of procedures for MA alarm management.

We have shown that PBRTQC is also achievable in laboratories whose LIS does not initially have PBRTQC options. Loh *et al.* have systematized the characteristics a LIS needs to possess to efficiently allow PBRTQC (*21*). Based on them, additional software solutions have been developed that enabled the application of MA procedures in our laboratory. In our case, crucial to the successful implementation of PBRTQC in the LIS were: the detailed recommendations of the IFCC group, the proactive attitude of biochemists in adapting these recommendations to local requirements, and the cooperation of software developers who recognized the importance of adding these options to the LIS (*21*).

Finally, after successfully selecting and implementing MA control procedures in the LIS, it is necessary to define a strategy for managing MA alarms. In developing an algorithm for the MA alarms work-up, we were guided by the experiences, solutions, and recommendations of other authors (*2*, *5*, *9*, *20*). However, we had to adapt these guidelines to the situation in the particular laboratory. Since our results showed that 65% of MA alarms were caused either by an individual pathological finding or a pre-analytical problem in the sample, it seemed fully justified to start the alarm evaluation by reviewing patient results before the alarm, as recommended by Badrick *et al.* (*5*). The LIS option that allows excluding a patient with a chronic pathological value of a parameter from future MA calculations, previously employed by other authors, has also shown to be useful in our laboratory (*9*). Repeated testing on another analyser as one of the steps towards finding the cause of the alarm was not an option for us, as we only have one clinical chemistry analyser (*9*). Agreeing that an analysis of internal quality control alone is not enough to evaluate the MA alarm, we decided on the approach described by Liu *et al.* involving the re-analysis of patient samples from a stable period on the same analyser (*5*, *20*).

The average frequency of MA alarms in our study did not lead to alarm fatigue, which is one of the key requirements for incorporating MA in everyday laboratory practice (*5*, *22*). An average number of less than one alarm *per* week is manageable even in a laboratory with a small number of biochemists. Therefore, we did not make any additional adjustments to the MA settings that would increase the specificity but decrease the sensitivity of this control instrument (*5*). Of course, during the routine application of MA procedures, some fine-tuning may be required based on the observed performance of this tool. The percentage of MA alarm occurrence in relation to the total generated number of MA values was in agreement with the results published by other authors (*7*, *23*). As in the work of van Rossum, MA alarms most often occurred on sodium (*23*). Notably, there were no violations of the control rule of traditional quality control that would require corrective measures, though small shifts on the Levey-Jennings chart were detected twice compared to the previous control measurement. This may indicate that, for some tests, PBRTQC is more sensitive in detecting bias than traditional quality control. The clinical significance of these biases, on the other hand, is debatable, especially in a primary care laboratory (*23*). At the same time, we agree with the observation of Ng *et al.* that it should be examined whether more frequent recalibration of the sodium test (more than the reagent manufacturer’s minimal recommendation) would reduce the frequency of MA alarms on this test (*24*).

When discussing the selection of TEa as the critical bias, we followed the recommendations of the IFCC Working group on PBRTQC. They state that the optimization and validation of PBRTQC procedures are often based on reliable TEa detection (*19*, *25*). In addition, in several papers dealing with control techniques, TEa is considered the cut-off value for detecting clinically significant error (*2*, *8*, *9*, *16*, *26*). When we talk about selecting TEa from different sources, we used CLIA data because we have already used it for calculating Sigma metrics in our laboratory. There are, of course, more stringent specifications of analytical performance goals, but other researchers have already concluded that MA procedures cannot detect biological variation-based TEa (*9*, *15*, *17*). Nevertheless, we checked this, and found that minimum TEa based on biological variation could be detected by our optimized MA procedures within the daily test production – always for AST and cholesterol and in about 50% of cases for albumin, total protein and sodium (for the last two, only for positive bias).

Overall, our results showed that a small-volume laboratory working with the general population without specific pathologies is suitable for the implementation of PBRTQC because, due to the homogeneity of patient results, even small individual shifts are a trigger for MA alarms. At the same time, the MA control has successfully detected pre-analytical problems and this is an advantage of PBRTQC over traditional quality control (*10*). PBRTQC procedures control both the pre-analytical and the analytical phase (*5*). In addition, we already know from the literature that PBRTQC alarms can be caused by extreme results of individual patients (*5*). The results of our work are in agreement with these literature data, and we do not consider them false alarms but proof that MA control procedures are capable of detecting different types of laboratory errors (*10*, *22*). We believe the demonstrated sensitivity of PBRTQC provides additional security by enabling rapid detection of any situation that deviates from the usual, whether caused by a pre-analytical or analytical problem. Also, we agree with the other authors that, although it has advantages, PBRTQC cannot replace the traditional quality control, but rather supplement and strengthen it (*2*, *5*, *10*). Combining MA procedures with traditional quality control into a single quality control plan yielding more reliable results is yet to be done in our laboratory. However, data from other authors’ articles indicating cost and time savings suggest it is worth investing in the MA alarm work-up protocols (*27*). Of course, in the near future, we can expect machine-learning tools to largely eliminate the need for laboratory specialists’ work in this area (*10*).

It is quite clear that global efforts to promote the idea of PBRTQC make complete sense only when they are locally accepted in as many laboratories as possible around the world (*5*, *11*, *19*, *23*). We believe the paper represents a contribution to this global idea and can assist in changing the paradigm of traditional quality control in our country (*16*, *27*).

Regarding the limitations of our study, the first one concerns the fact that implementation was performed on 10 clinical chemistry tests whose MA procedures had the best bias detection capabilities during the optimization process and the selection of tests should be based on a risk-based quality control plan. Secondly, the benefit of using MA procedures as an additional quality control tool should be demonstrated by estimating the number of samples in which the release of an inaccurate result would be prevented due to the detection of bias between two regular quality control measurements.

In conclusion, this paper showed it is possible, even in a laboratory with a small testing volume, to successfully select MA procedures, implement them in the LIS, and use them for continuous analytical quality control. Review of patient results by biochemists, re-analysis of patient samples from the stable period, analysis of internal quality control samples, and a review of analyser malfunctions and maintenance datalog are some of the proposals for an algorithm for managing MA alarms. Further research should focus on combining the described MA procedures and the existing traditional control tools into a laboratory quality control plan based on risk.
